# Hypothalamic Perineuronal Nets Are Regulated by Sex and Dietary Interventions

**DOI:** 10.3389/fphys.2021.714104

**Published:** 2021-07-28

**Authors:** Nan Zhang, Zili Yan, Hailan Liu, Meng Yu, Yang He, Hesong Liu, Chen Liang, Longlong Tu, Lina Wang, Na Yin, Junying Han, Nikolas Scarcelli, Yongjie Yang, Chunmei Wang, Tianshu Zeng, Lu-Lu Chen, Yong Xu

**Affiliations:** ^1^Children’s Nutrition Research Center, Department of Pediatrics, Baylor College of Medicine, Houston, TX, United States; ^2^Department of Endocrinology, Union Hospital, Tongji Medical College, Huazhong University of Science and Technology, Wuhan, China; ^3^Hubei Provincial Clinical Research Center for Diabetes and Metabolic Disorder, Wuhan, China; ^4^Department of Molecular and Cellular Biology, Baylor College of Medicine, Houston, TX, United States

**Keywords:** hypothalamus, sex difference, diet, perineuronal net, feeding, glucose

## Abstract

Perineuronal nets (PNNs) are widely present in the hypothalamus, and are thought to provide physical protection and ion buffering for neurons and regulate their synaptic plasticity and intracellular signaling. Recent evidence indicates that PNNs in the mediobasal hypothalamus play an important role in the regulation of glucose homeostasis. However, whether and how hypothalamic PNNs are regulated are not fully understood. In the present study, we examined whether PNNs in various hypothalamic regions in mice can be regulated by sex, gonadal hormones, dietary interventions, or their interactions. We demonstrated that gonadal hormones are required to maintain normal PNNs in the arcuate nucleus of hypothalamus in both male and female mice. In addition, PNNs in the terete hypothalamic nucleus display a sexual dimorphism with females higher than males, and high-fat diet feeding increases terete PNNs only in female mice but not in male mice. On the other hand, PNNs in other hypothalamic regions are not influenced by sex, gonadal hormones or dietary interventions. In summary, we demonstrated that hypothalamic PNNs are regulated in a region-specific manner and these results provide a framework to further investigate the potential functions of PNNs in regulating energy/glucose homeostasis at the interplay of sex, gonadal hormones and diets.

## Introduction

Obesity is now recognized as a serious global health problem due to its increasing prevalence and comorbidities, e.g., the metabolic syndrome. The World Health Organization reported that over 650 million adults worldwide were obese in 2016 and 40 million children under the age of 5 were overweight or obese in 2018. In United States, the prevalence of adult obesity was 42.4% in 2017∼2018 according to the Centers for Disease Control and Prevention. The etiology of human obesity is not fully understood, and effective treatments for obesity and associated metabolic disorders are limited. Recent studies revealed genetic and epigenetic bases for variations in human body mass index (BMI) (Bliss and Collingridge, 1993; Lerner et al., 2015; Chan et al., 2017), and strikingly the majority of BMI-associated genetic variants affect genes that are enriched in the brain (Locke et al., 2015; Turcot et al., 2018). In particular, the brain hypothalamus receives metabolic and/or hormonal signals reflecting the body’s nutritional status and coordinates neuroendocrine and behavioral responses to maintain body weight balance (Hetherington and Ranson, 1940; Elmquist et al., 1999; Morton et al., 2006). Indeed, various genetic variants associated with human obesity have been demonstrated to cause energy and/or glucose dysregulations through impairing functions of neurons and/or neurocircuits within the hypothalamus (Yang and Xu, 2021).

Perineuronal nets (PNNs) in the brain are condensed glycosaminoglycan-rich extracellular matrix structures (Testa et al., 2019). PNNs typically enmesh neurons in defined circuits, and are thought to provide physical protection and ion buffering for neurons, and regulate their synaptic plasticity and intracellular signaling (Testa et al., 2019). PNNs abundantly exist in the forebrain regions, e.g., the cortex and the hippocampus, and the PNNs levels in these regions can be regulated by animals’ experience, and have been implicated in various neurobiological disorders such as schizophrenia, bipolar disorder, Alzheimer’s disease and addictions (Testa et al., 2019). Recent evidence indicates that chemical disruption of PNNs in the mediobasal hypothalamus significantly blunts the glucose-lowering effects of central action of fibroblast growth factor 1 (FGF1) in obese Zucker diabetic fatty (ZDF) rats (Alonge et al., 2020). Thus, hypothalamic PNNs may also play important roles in the regulation of energy and/or glucose homeostasis. Indeed, abundant PNNs are present in multiple hypothalamic regions (Horii-Hayashi et al., 2015; Mirzadeh et al., 2019), but whether and how these hypothalamic PNNs are regulated are not fully understood.

The PNNs in the prefrontal cortex and hippocampus have been reported to be influenced by obesogenic dietary challenges (Dingess et al., 2018; Reichelt et al., 2019, 2021); interestingly, sex differences were observed in PNNs in the prefrontal cortex (Dingess et al., 2020; Gildawie et al., 2020). Thus, in the present study, we sought to examine whether PNNs in various hypothalamic regions can be regulated by dietary interventions in mice. Since many hypothalamic regions are sexually dimorphic and/or regulated by gonadal hormones (Wang and Xu, 2019; Kammel and Correa, 2020; Liu et al., 2021), we also explored the potential effects of sex and/or gonadal hormones on PNN levels. Our results revealed that sex, gonadal hormones and/or dietary interventions can regulate PNNs in a region-specific manner and provided a framework to further investigate the potential functions of PNNs in regulating energy/glucose homeostasis at the interplay of sex, gonadal hormones and diets.

## Materials and Methods

### Study Animals

C57BL/6J male and female mice were group-housed, respectively, until 14 weeks of age in a temperature-controlled room under a 12:12 h light-dark cycle with ad lib access to regular chow diet (5V5R-Advanced Protocol PicoLab Select Rodent 50 IF/6F, PicoLab) and water. Forty nine mice were divided into 16 groups by four factors (1) sex: male or female groups; (2) surgeries: receiving sham or castration (CAST)/ovariectomy (OVX) surgeries at 14 weeks of age (see below); (3) dietary interventions: chronic feeding with chow or a high-fat diet (HFD, D12492i Rodent Diet With 60 kcal% Fat, Research Diets, Inc.), (4) nutritional status: fed or fasted at the time of perfuse (see below).

Penk-IRES2-Cre mice were purchased from Jackson Laboratory (#025112) which express Cre recombinase selectively in preproenkephalin (PENK) neurons (Harris et al., 2019; Heinsbroek et al., 2020). These mice were bred with Rosa26-LSL-tdTOMATO mice (Madisen et al., 2010) (Jackson Laboratory, #007905) to generate Penk-IRES2-Cre/Rosa26-LSL-tdTOMATO reporter mice, in which PENK neurons are labeled by tdTOMATO. These mice were used to examine whether PNNs enmeshed PENK-expressing neurons, as described below.

Care of all animals and procedures were approved by the Baylor College of Medicine Institutional Animal Care and Use Committee.

### Surgery

At the age of 14 weeks, either bilateral gonadectomy (CAST for males and OVX for females) or sham surgeries were executed in these mice. The mice were injected with sustained-release buprenorphine 1 mg/kg and meloxicam 4 mg/kg before the surgery and meloxicam 4 mg/kg each day for 3 days after the surgery as analgesics. The mice were anesthetized with inhaled 2% isoflurane during the surgery. For OVX in females, the dorsolateral incisions (2 cm) were made, followed by blunted separation of the subcutaneous fat, and incision (1.5 cm) the abdominal wall to expose the reproductive tract. The ovaries were isolated and excised. The reproductive tract was returned to the peritoneum and the incisions on the abdominal wall were sutured using sterile Vicryl thread (Coated VICRYL^®^ (polyglactin 910) Sutures, 5-0, 95057-126); the skin incisions were sutured by sterile Nylon thread (ETHILON^®^ Nylon Sutures, Black Monofilament, Pliabilized, 95056-900). For sham surgeries in female mice, the procedures were the same, except that the ovaries were kept intact. For castration in males, a central vertical incision (3 cm) was made, followed by blunted separation of any subcutaneous fat to locate the vas deferens. The vas deferens were ligated with Vicryl; the testes were cut and taken away from the fat pad. The skin incisions were sutured by sterile Nylon thread. For sham surgeries in male mice, the procedures were the same, except that the testes were kept intact.

### Food Intake, Body Weight, Glucose, and Body Composition

One week after the surgeries, mice were randomly divided into two groups to be fed either regular chow or HFD *ad libitum* for 4 weeks. Food intake, body weight and fed glucose were measured before the surgeries and once every week afterward for 5 weeks. The glucose was measured using the glucometer *via* the tail vein; to avoid confounding effects from circadian clock and feeding behavior on the glucose level, we always measured blood glucose in the morning of the day and after a 2-h short fasting to ensure the empty stomach. Body composition (fat mass and lean mass) was examined with the Bruker minispec mq10 MRS system before the surgeries and 5 weeks afterward.

### Perfusion and WFA Staining

Five weeks after the surgeries, mice were further divided into fasted and *ad libitum* groups before the perfusion. For the fasted group, mice were fasted overnight and then perfused in the next morning; for the *ad libitum* group, both food and water were provided *ad libitum* at all time before the perfusion. For the perfusion, mice were anesthetized with inhaled isoflurane and perfused with saline followed by 10% formalin (10% Neutral Buffered Formalin, 16004-128, VWR). Since we needed to perfuse mice at the exactly same time point after the surgery, we could not guarantee that female sham mice were perfused at the same estrous stage. Given this limitation, we recorded the estrous stage of each female sham mouse at the time of the perfusion. Notably, female sham mice in different diet or nutritional subgroups had similar patterns of estrous cycle with at least two different stages (Supplementary Table 1). In addition, we failed to observe any significant effects of the estrous stage on the WFA signals by the Kruskal–Wallis one-way ANOVA analysis (Supplementary Tables 2, 3).

The brain sections were cut at 25 μm and collected into five consecutive series. One series is used for the following staining and quantification. Free-floating tissue sections were incubated overnight at room temperature for 22 h using a 1:500 dilution of biotin-labeled Wisteria floribunda agglutinin (WFA; L1516, Sigma-Aldrich) in PBS + 0.25% Triton X-100. The WFA staining has been validated by digesting PNNs using chondroitinase ABC and an ultrastructural analysis of WFA-diaminobenzidine labeling in a previous study (Mirzadeh et al., 2019). The sections were then washed and incubated for 2 h at room temperature in 1:500 Streptavidin protein DyLight 488 (21832, Invitrogen). The sections were mounted on slides and coverslipped with the DAPI-containing mounting medium (H-1500; Vector Laboratories). Images were captured using a fluorescence microscope with 1-second exposure time and no change of contrast for all pictures to visualize various hypothalamic nuclei, including the arcuate hypothalamic nucleus (ARH), the terete hypothalamic nucleus (TE), the paraventricular hypothalamic nucleus (PVH), the lateral hypothalamus (LH), the anterior hypothalamus (AH), the ventromedial hypothalamic nucleus (VMH), the dorsomedial hypothalamic nucleus (DMH), and the perifornical area of the anterior hypothalamus (PeFAH) within 4 days after the staining to avoid the confounding effects of fluorescence quenching. The experimenters who quantified the sections were blinded to which manipulations were performed to the mice. Fluorescence intensity of PNNs for each image was quantified using an established method in ImageJ by the macro plugin “Perineuronal net Intensity Program for the Standardization and Quantification of ECM Analysis” (PIPSQUEAK AI v5.3.9, Rewire Neuro, Inc.) following the region of interest (“ROI”) method (Slaker et al., 2016). Briefly, the Rolling Ball Radius function of ImageJ, which removes smooth continuous background, was first used to remove variability in background staining across the image (Sternberg, 1983). A ROI was then created manually within four regions of an image lacking a PNN, and the average intensity and standard deviation (SD) of each of these ROIs were determined using ImageJ. Higher value ROI was selected to represent the background. To distinguish the PNN structure from general “loose” extracellular matrix (ECM) staining, a threshold was set at two SD above the background, and only signals above this threshold was considered PNN and taken into quantification. All pixels below this threshold were set to “not a number.” Then, the ROI was created if PNNs surrounded at least 2/3 of the soma. Both the number of PNN-enmeshed cells and the average intensity of PNNs were recorded and calculated by PIPSQUEAK AI.

In order to explore the neurochemical identity of PNN-enmeshed neurons, we performed WFA staining in Penk-IRES2-Cre/Rosa26-LSL-tdTOMATO mice as described above. We also co-stained WFA with estrogen receptor α (ERα), androgen receptor (AdR), or calcitonin gene-related peptide (CGRP). Briefly, sections were first incubated in WFA (1:1,000) in PBS with 0.25% Triton X-100 for 22 h at room temperature, followed by Streptavidin protein DyLight 488 (1:500) for 2 h at room temperature. Then after blocking in 3% donkey normal blocking serum (300 μl donkey normal serum in 10 ml PBT) for 2 h, sections were incubated in primary for 24 h and secondary antibodies for 2 h in room temperature in PBS/0.25% Triton X-100 and 3% donkey normal blocking serum. Primary antibodies we used include the rabbit anti-ERα antibody (1:5,000 in 4°C for 24 h; 06-935, Millipore) (Cao et al., 2014; Xu et al., 2015), the rabbit anti-AdR antibody (1:200 in room temperature for 22 h; 06-680, Sigma-Aldrich) (Ghatge et al., 2005) and the rabbit anti-CGRP pAb (1:500 in 4°C for 24 h; A5542, ABclonal) (Zhao et al., 2019). The secondary antibody was donkey anti-rabbit (1:1,000, Alexa Fluor Plus 594, A32754; Invitrogen). The sections were mounted on slides, coverslipped with the DAPI-containing mounting medium and imaged using Zeiss Axio Scan. Z1.

### Statistics

Statistical analysis was done using SPSS (SPSS 22.0.0.0, IBM SPSS Statistics) and GraphPad Prism (GraphPad Prism 8.4.2, Graphpad software, LLC) unless otherwise indicated. As we have 3–4 factors to investigate, the multivariate linear regression was used to first investigate the independent influence of each factor on endpoints. This method can not only give a much clearer result of the influence of the factors comparing to ANOVA, but also adjust the confounding factors and give an independent effect of the factor. Specifically, the multivariate linear regression in SPSS was used to investigate the influence of 3 factors, namely sex, GDX and chronic dietary intervention, on various metabolic parameters (cumulative food intake, body weight change, blood glucose change, fat change and lean change). If any two factors were found to have significant effects, the *p* value for the interaction of these two factors was tested. For the PNN fluorescence intensity and the number of PNN-enmeshed cells, in addition to the sex, GDX and chronic diet intervention, we introduced a fourth factor, nutritional status, as mice were perfused either after an overnight fasting or *ad libitum*. Similarly, we first used the multivariate linear regression analyses in SPSS to examine effects of four factors on the PNN fluorescence intensity and the number of PNN-enmeshed cells in each region. When a significant factor was found, we then used graphs to further illustrate the results. Notably, as the male and the female had different ways of GDX surgery, the effect of GDX on PNN fluorescence intensity and the number of PNN-enmeshed cells was further tested in each sex when GDX showed a significant effect. Similarly, when the sex factor was found to have a significant effect on PNN fluorescence intensity and the number of PNN-enmeshed cells, the effect of sex was further tested in GDX and sham group individually. *P* < 0.05 was considered statistically significant.

In Supplementary Tables 4–8, variables were expressed as mean and SD if they were normally distributed. Variables were expressed as medians and interquartile ranges if they were not normally distributed. Analyses were performed using EmpowerStats (X&Y solutions, Boston, MA, United States^1^) and R^2^.

### Study Approval

Care of all animals and procedures were approved by the Baylor College of Medicine Institutional Animal Care and Use Committee.

## Results

### Distribution of WFA-Labeled PNNs in the Mouse Hypothalamus

**FIGURE 1:**
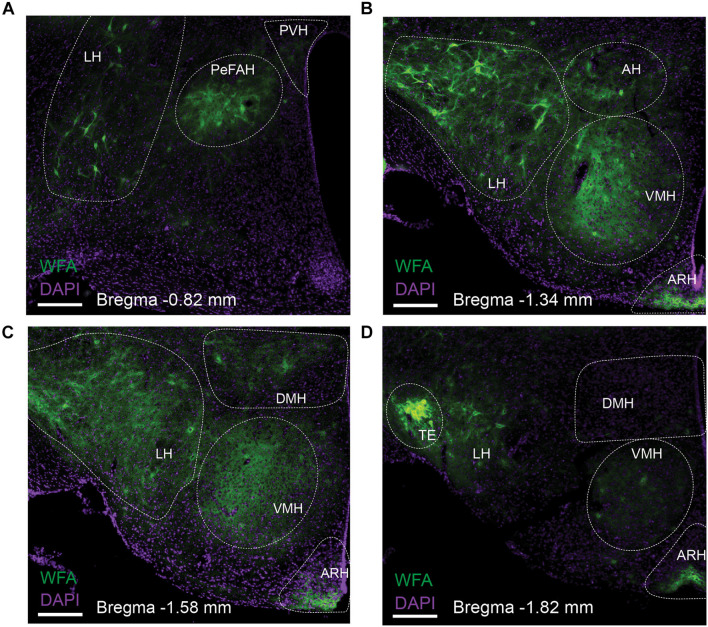
Distribution of WFA-labeled PNNs in the mouse hypothalamus. Representative fluorescent microscopic images showing WFA-labeled PNNs (green) and DAPI counterstaining (purple) in a series of coronal brain sections at the level of –0.82 mm **(A)**, –1.34 mm **(B)**, –1.58 mm **(C)** and –1.82 mm **(D)** relative to the Bregma. Scale bars = 200 μm. AH, the anterior hypothalamus; ARH, the arcuate hypothalamic nucleus; DMH, the dorsomedial hypothalamic nucleus; LH, the lateral hypothalamus; PeFAH, the perifornical area of the anterior hypothalamus; PVH, the paraventricular hypothalamic nucleus; TE, the terete hypothalamic nucleus; VMH, the ventromedial hypothalamic nucleus.

We first surveyed the distribution of PNNs in the mouse hypothalamus using WFA staining. Abundant WFA-labeled PNNs were detected in multiple hypothalamic regions, including the LH, the ARH, the VMH and the TE (Figure 1). In addition, we also observed modest WFA signals in the AH, the PVH and the DMH (Figure 1). Consistent with a previous report (Horii-Hayashi et al., 2015), WFA-labeled PNNs were also observed in a newly identified hypothalamic area, namely the PeFAH (Figure 1A). In line with this earlier finding (Horii-Hayashi et al., 2015), we observed that a portion of PNN-enmeshed PeFAH neurons are PENK-positive neurons (Supplementary Figures 1A,E). We also explored the neurochemical identity of PNN-enmeshed neurons in the ARH and TE. In the ARH, a portion of PNN-enmeshed neurons express ERα (Supplementary Figures 1B,F); interestingly, the majority of these neurons express androgen receptor (Supplementary Figures 1C,G). In the TE, we found that a portion of PNN-enmeshed neurons express CGRP (Supplementary Figures 1D,H).

### Effects of Sex, Gonadal Hormones and Dietary Interventions on Metabolic Parameters

Given the essential roles of the hypothalamus in regulating energy homeostasis, we sought to examine potential effects of dietary interventions on the level of PNNs in the hypothalamus. In order to explore potential influence by sex and/or gonadal hormones, we included both male and female mice with or without GDX in these analyses. Briefly, male and female C57BL/6J mice underwent sham or GDX (castration for males and ovariectomy for females) at the 14 weeks of age, followed by 4-week chronic feeding with either a regular chow diet or HFD. The metabolic parameters including calorie intake, body weight change, blood glucose change, fat change and lean change in various groups were shown in Supplementary Tables 4–7. In order to examine the independent effects of sex, GDX, dietary interventions on these metabolic parameters, we used the multivariate linear regression analysis. As expected, the multivariate linear regression analysis revealed that compared to chow-fed groups, HFD feeding had profound effects on multiple metabolic parameters, including significantly increasing calorie intake, blood glucose, body weight gain and fat mass gain (Table 1). Notably, fat mass gain was significantly smaller in females than in males (Table 1). We also noted that lean mass gain was significantly larger in females than in males, and that GDX significantly reduced lean mass gain; in addition, there was a significant interaction between sex and GDX on this parameter (Table 1). In summary, multiple metabolic parameters in mice were influenced by sex, gonadal hormones, the dietary interventions, and/or their interactions, as reported by others (Moverare-Skrtic et al., 2006; Riant et al., 2009).

**TABLE 1:** Effects of sex, GDX, and diet on various metabolic parameters.

	B	*p* value	95.0% Confidence interval	
			Lower bound	Upper bound
**Cumulative intake (calorie)**				
Female vs Male^#^	–13.234	0.298	–38.542	12.075
GDX vs Sham^##^	7.461	0.554	–17.798	32.721
High fat diet vs Regular chow^###^	56.660	*p*< 0.001***	31.306	82.013
Body weight change (g)				
Female vs Male^#^	–0.448	0.620	–2.255	1.359
GDX vs Sham^##^	0.532	0.556	–1.275	2.339
High fat diet vs Regular chow^###^	5.389	*p*< 0.001***	3.582	7.196
**Blood glucose change (mg/dL)**				
Female vs Male^#^	13.141	0.128	–3.915	30.198
GDX vs Sham^##^	1.143	0.893	–15.913	18.200
High fat diet vs Regular chow^###^	37.838	*p*< 0.001***	20.782	54.894
**Fat change (g)**				
Female vs Male^#^	–2.052	0.012*	–3.633	–0.471
GDX vs Sham^##^	1.495	0.063	–0.085	3.076
High fat diet vs Regular Chow^###^	5.539	*p*< 0.001***	3.958	7.120
*P* for the interaction of Sex and Diet		0.226		
**Lean change (g)**				
Female vs Male^#^	1.405	*p*< 0.001***	0.828	1.983
GDX vs Sham^##^	–0.805	0.007**	–1.383	–0.228
*P* for the interaction of Sex and GDX		*p*< 0.001***		
High fat diet vs Regular chow^###^	–0.107	0.710	–0.684	0.470

^#^Here we use male mice as reference. ^##^Here we use sham mice as reference. ^###^Here we use regular chow as reference. B stands for regression coefficients. The p value is the smallest level α0 such that we would reject the null-hypothesis at level α0 with the observed data. The confidence interval (CI) is a range of values that’s likely to include a population value with a certain degree of confidence. *p < 0.05, **p < 0.01, ***p < 0.001.

### Effects of Gonadal Hormones on PNN in the ARH

**FIGURE 2:**
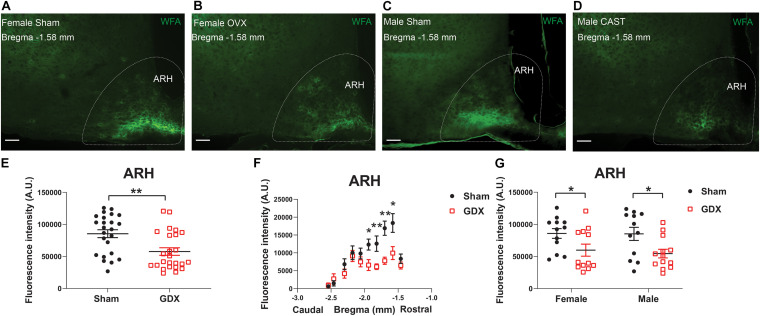
GDX reduces PNNs in the ARH. **(A–D)** Representative fluorescence microscopic images showing WFA-labeled PNNs (green) in the ARH of female sham **(A)**, female OVX **(B)**, male sham **(C)** and male CAST mice **(D)**. Scale bars = 50 μm. ARH, the arcuate hypothalamic nucleus. **(E)** Quantification of the total WFA fluorescence intensity in the ARH from sham vs GDX mice. *N* = 24 or 25 mice per group. Data are presented with mean ± SEM with individual data points. **P < 0.01 in unpaired two-tailed Student’s *t*-tests. **(F)** Quantification of the WFA fluorescence intensity at various rostral-caudal levels of the ARH from sham vs GDX mice. *N* = 24 or 25 mice per group. Data are presented with mean ± SEM. *P < 0.05 and **P < 0.01 in unpaired two-tailed Student’s *t-*tests at each level. *P* < 0.0001 for sham vs GDX mice in 2-way ANOVA with repeated measures. **(G)** Quantification of the total WFA fluorescence intensity in the ARH from female or male mice with sham or GDX surgeries. *N* = 12 or 13 mice per group. Data are presented with mean ± SEM with individual data points. *P < 0.05 in two-way ANOVA analysis followed by Holm-Sidak *post hoc* tests.

At the end of 4-week feeding, mice were then perfused either *ad libitum* or after an overnight fasting. We then used these fixed brain samples for WFA staining and quantified the level of WFA-labeled PNNs in each hypothalamic region in various conditions. The WFA signals in these hypothalamic regions in various groups were depicted in Supplementary Tables 2–6. Multivariate linear regression analyses were applied to reveal the independent effects of these factors on the WFA signals in different hypothalamic regions. In the ARH, the WFA fluorescence intensity and the number of PNN-enmeshed cells were not significantly affected by chronic HFD feeding (vs chow), or by the acute fasting (vs *ad libitum*), as revealed by the multivariate linear regression analysis (Tables 2, 3). Interestingly, we noted that GDX significantly reduced WFA fluorescence intensity and the number of PNN-enmeshed cells in the ARH compared to the sham group (Tables 2, 3 and Figures 2A–E). A detailed analysis at different rostral-caudal levels revealed that GDX mainly reduced WFA fluorescence intensity in the rostral to medial ARH (−1.94 to −1.58 mm to Bregma) but the caudal ARH was not affected (Figure 2F). Because female and male mice experienced different GDX surgeries (OVX for females and CAST for males), we used a two-way ANOVA analysis to further analyze the interaction between sex and GDX. We found that the inhibitory effect of GDX was independent of sex; in other words, both OVX in females and CAST in males significantly reduced WFA fluorescence intensity in the ARH (Figure 2G). We also examined effects of dietary interventions on ARH PNNs in sham or in GDX groups, respectively, but did not detect any significant effect of by either chronic HFD feeding or by acute fasting (data not shown). Thus, these results indicate that endogenous gonadal hormones in both males and females are required to maintain normal PNNs in the ARH.

**TABLE 2:** Effects of sex, GDX, diet and fasting on PNN fluorescence intensity in the ARH.

	B	*p* value	95.0% Confidence interval	
			Lower bound	Upper bound
Female vs Male^#^	3,216.040	0.710	−14,091.082	20,523.162
GDX vs Sham^##^	−28,525.814	0.002**	−45,832.935	−11,218.692
High fat diet vs Regular chow^###^	5,478.986	0.527	−11,858.261	22,816.234
Fast vs Ab libitum^####^	1,070.635	0.902	−16,266.612	18,407.883

^#^Here we use male mice as reference. ^##^Here we use sham mice as reference. ^###^Here we use regular chow as reference. ^####^Here we use fed mice as reference. B stands for regression coefficients. The p value is the smallest level α0 such that we would reject the null-hypothesis at level α0 with the observed data. The confidence interval (CI) is a range of values that’s likely to include a population value with a certain degree of confidence. **p < 0.01.

**TABLE 3:** Effects of sex, GDX, diet, and fasting on the number of PNN-enmeshed cells in the ARH.

	B	*p* value	95.0% Confidence interval	
			Lower Bound	Upper Bound
Female vs Male^#^	1.572	0.826	−12.736	15.880
GDX vs Sham^##^	−15.322	0.036*	−29.630	−1.014
High fat diet vs Regular chow^###^	−0.470	0.948	−14.803	13.863
Fast vs Ab libitum^####^	6.222	0.386	−8.111	20.555

^#^Here we use male mice as reference. ^##^Here we use sham mice as reference. ^###^Here we use regular chow as reference. ^####^Here we use fed mice as reference. B stands for regression coefficients. The p value is the smallest level α0 such that we would reject the null-hypothesis at level α0 with the observed data. The confidence interval (CI) is a range of values that’s likely to include a population value with a certain degree of confidence. *p < 0.05.

### Effects of Sex and HFD Feeding on PNN in the TE

**FIGURE 3:**
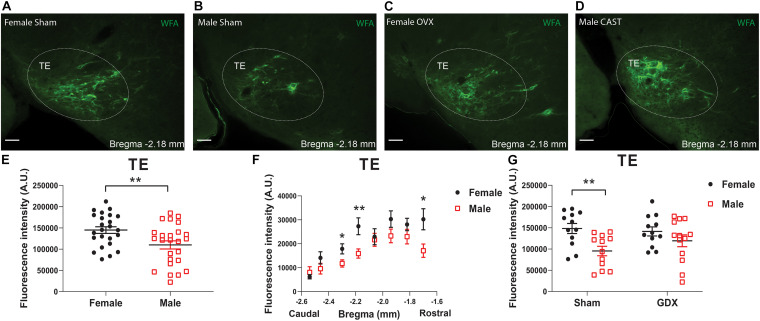
PNNs in the TE display a sexual dimorphism. **(A–D)** Representative fluorescence microscopic images showing WFA-labeled PNNs (green) in the TE of female sham **(A)**, male sham **(B)**, female OVX **(C)**, and male CAST mice **(D)**. Scale bars = 50 μm. TE, the terete hypothalamic nucleus. **(E)** Quantification of the total WFA fluorescence intensity in the TE from female vs male mice. *N* = 24 or 25 mice per group. Data are presented with mean ± SEM with individual data points. **P < 0.01 in unpaired two-tailed Student’s *t*-tests. **(F)** Quantification of the WFA fluorescence intensity at various rostral-caudal levels of the TE from female vs male mice. *N* = 24 or 25 mice per group. Data are presented with mean ± SEM. *P < 0.05 and **P < 0.01 in unpaired two-tailed Student’s *t-*tests at each level. *P* = 0.0002 for female vs male mice in 2-way ANOVA with repeated measures. **(G)** Quantification of the total WFA fluorescence intensity in the TE from female or male mice with sham or GDX surgeries. *N* = 12 or 13 mice per group. Data are presented with mean ± SEM with individual data points. **P < 0.01 in two-way ANOVA analysis followed by Sidak *post hoc* tests.

In the TE, female mice showed significantly higher WFA fluorescence intensity compared to males, as revealed by the multivariate linear regression analysis (Table 4 and Figures 3A–E), and this sex difference existed in a rostral subdivision (−1.70 mm to Bregma) and a medial-caudal subdivision (−2.18 to −2.30 mm to Bregma) of the TE (Figure 3F). Using a two-way ANOVA analysis, we further found that the significant sex difference only existed in sham mice, but not in GDX mice (Figure 3G). Notably, sex trended to influence the number of PNN-enmeshed TE cells, but this effect failed to reach statistical significance in the multivariate linear regression analysis (Table 5). Nevertheless, our results indicate that gonad-intact female mice have higher PNNs in the TE than gonad-intact males.

**TABLE 4:** Effects of sex, GDX, diet and nutritional status on PNN fluorescence intensity in the TE.

	B	*p* value	95.0% Confidence interval	
			Lower Bound	Upper Bound
Female vs Male^#^	37,265.520	0.004**	12,278.584	62,252.456
GDX vs Sham^##^	8,878.365	0.478	−16,108.572	33,865.301
High fat diet vs Regular chow^###^	3,443.149	0.783	−21,587.281	28,473.579
Fast vs Ab libitum^####^	−2,328.269	0.852	−27,358.698	22,702.161

^#^Here we use male mice as reference. ^##^Here we use sham mice as reference. ^###^Here we use regular chow as reference. ^####^Here we use fed mice as reference. B stands for regression coefficients. The p value is the smallest level α0 such that we would reject the null-hypothesis at level α0 with the observed data. The confidence interval (CI) is a range of values that’s likely to include a population value with a certain degree of confidence. **p < 0.01.

**TABLE 5:** Effects of sex, GDX, diet and nutritional status on the number of PNN-enmeshed cells in the TE.

	B	*p* value	95.0% Confidence interval	
			Lower Bound	Upper Bound
Female vs Male^#^	8.148	0.089	−1.300	17.597
GDX vs Sham^##^	4.935	0.298	−4.514	14.383
High fat diet vs Regular chow^###^	3.740	0.430	−5.725	13.205
Fast vs Ab libitum^####^	−5.337	0.262	−14.802	4.128

*^#^Here we use male mice as reference. ^##^Here we use sham mice as reference. ^###^Here we use regular chow as reference. ^####^Here we use fed mice as reference. B stands for regression coefficients. The p value is the smallest level α0 such that we would reject the null-hypothesis at level α0 with the observed data. The confidence interval (CI) is a range of values that’s likely to include a population value with a certain degree of confidence.*

**FIGURE 4:**
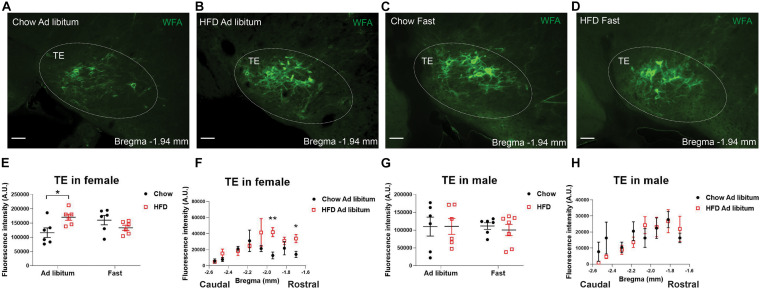
HFD feeding increases TE PNNs in female mice. **(A–D)** Representative fluorescence microscopic images showing WFA-labeled PNNs (green) in the TE of female mice that were fed chow *ad libitum* **(A)**, HFD *ad libitum* sham **(B)**, chow after an overnight fasting **(C)**, and HFD after an overnight fasting **(D)**. Scale bars = 50 μm. TE, the terete hypothalamic nucleus. **(E)** Quantification of the total WFA fluorescence intensity in the TE from female mice with various dietary interventions. *N* = 6 mice per group. Data are presented with mean ± SEM with individual data points. *P < 0.05 in two-way ANOVA analysis followed by Sidak *post hoc* tests. **(F)** Quantification of the WFA fluorescence intensity at various rostral-caudal levels of the TE from female mice fed with chow or HFD *ad libitum*. *N* = 6 mice per group. Data are presented with mean ± SEM. *P < 0.05 and **P < 0.01 in unpaired two-tailed Student’s *t*-tests at each level. *P* = 0.0133 for chow vs HFD *ad libitum* female mice in 2-way ANOVA with repeated measures. **(G)** Quantification of the total WFA fluorescence intensity in the TE from male mice with various dietary interventions. *N* = 6 or 7 mice per group. Data are presented with mean ± SEM with individual data points. No significance was detected in 2-way ANOVA analysis. **(H)** Quantification of the WFA fluorescence intensity at various rostral-caudal levels of the TE from male mice fed with chow or HFD *ad libitum*. *N* = 6 mice per group. Data are presented with mean ± SEM.

Given the clear influence by sex, we reanalyzed the effects of HFD feeding in each sex individually and used a two-way ANOVA analysis followed by Sidak tests to further explore potential interactions between chronic HFD feeding and acute fasting. In female mice fed *ad libitum*, chronic HFD feeding significantly enhanced WFA fluorescence intensity in the TE compared to chow-fed females, but this effect was not observed after an overnight fasting (Figures 4A–E). This HFD-induced increases existed in a rostral-medial subdivision (−1.70 to −1.94 mm to Bregma) of the TE (Figure 4F). On the other hand, in male mice, neither chronic HFD feeding nor acute fasting induced any changes in WFA fluorescence intensity in the TE (Figures 4G,H). Thus, these results indicate that chronic HFD feeding can increase PNNs in the TE in female mice but not in male mice.

### Other Hypothalamic Regions

We also analyzed effects of sex, gonadal hormones, and/or dietary interventions on WFA signals in all other hypothalamic regions. The multivariate linear regression analysis revealed that WFA fluorescence intensity and the number of PNN-enmeshed cells in the PVH, the PeFAH, the LH, the AH, the VMH, or the DMH, were not significantly altered by these conditions (Tables 6, 7).

**TABLE 6:** Effects of sex, GDX, diet and nutritional status on PNN fluorescence intensity in the PVH, PeFAH, LH, AH, VMH, and DMH.

	B	*p* value	95.0% Confidence interval	
			Lower Bound	Upper Bound
**PVH**				
Female vs Male^#^	1,093.487	0.785	−6,950.901	9,137.874
GDX vs Sham^##^	1,801.150	0.649	−6,123.285	9,725.586
High fat diet vs Regular chow^###^	7,111.050	0.082	−948.109	15,170.209
Fast vs Ab libitum^####^	1,437.498	0.721	−6,621.661	9,496.657
**PeFAH**				
Female vs Male^#^	10,127.575	0.447	−16,501.677	36,756.827
GDX vs Sham^##^	12,003.295	0.361	−14,228.883	38,235.473
High fat diet vs Regular chow^###^	12,968.496	0.332	−13,709.655	39,646.646
Fast vs Ab libitum^####^	3,879.818	0.771	−22,798.333	30,557.969
**LH**				
Female vs Male^#^	48,723.960	0.118	−12,839.004	110,286.924
GDX vs Sham^##^	25,737.089	0.397	−34,907.897	86,382.074
High fat diet vs Regular chow^###^	−22,043.519	0.475	−83,719.530	39,632.491
Fast vs Ab libitum^####^	−17,950.520	0.560	−79,626.531	43,725.490
**AH**				
Female vs Male^#^	9,830.509	0.347	−11,017.746	30,678.764
GDX vs Sham^##^	6,991.586	0.496	−13,545.796	27,528.969
High fat diet vs Regular chow^###^	6,079.521	0.560	−14,807.017	26,966.059
Fast vs Ab libitum^####^	599.040	0.954	−20,287.498	21,485.578
**VMH**				
Female vs Male^#^	8,833.935	0.639	−28,921.823	46,589.693
GDX vs Sham^##^	6,705.915	0.718	−30,486.859	43,898.688
High fat diet vs Regular chow^###^	−14,866.204	0.432	−52,691.292	22,958.884
Fast vs Ab libitum^####^	−19,095.426	0.314	−56,920.514	18,729.662
**DMH**				
Female vs Male^#^	−2,075.195	0.135	−4,826.375	675.985
GDX vs Sham^##^	267.179	0.843	−2,442.978	2,977.336
High fat diet vs Regular chow^###^	1,587.169	0.252	−1,169.064	4,343.401
Fast vs Ab libitum^####^	−487.298	0.723	−3,243.531	2,268.934

*^#^Here we use male mice as reference. ^##^Here we use sham mice as reference. ^###^Here we use regular chow as reference. ^####^Here we use fed mice as reference. B stands for regression coefficients. The p value is the smallest level α0 such that we would reject the null-hypothesis at level α0 with the observed data. The confidence interval (CI) is a range of values that’s likely to include a population value with a certain degree of confidence.*

**TABLE 7:** Effects of sex, GDX, diet and nutritional status on the number of PNN-enmeshed cells in the PVH, PeFAH, LH, AH, VMH, and DMH.

	B	*p* value	95.0% Confidence interval	
			Lower Bound	Upper Bound
**PVH**				
Female vs Male^#^	0.144	0.960	−5.675	5.963
GDX vs Sham^##^	−2.477	0.396	−8.296	3.341
High fat diet vs Regular chow^###^	2.137	0.464	−3.692	7.966
Fast vs Ab libitum^####^	−0.632	0.828	−6.461	5.196
**PeFAH**				
Female vs Male^#^	−3.606	0.535	−15.233	8.021
GDX vs Sham^##^	6.606	0.258	−5.021	18.233
High fat diet vs Regular chow^###^	2.535	0.663	−9.112	14.182
Fast vs Ab libitum^####^	−8.850	0.133	−20.497	2.798
**LH**				
Female vs Male^#^	10.649	0.299	−9.781	31.078
GDX vs Sham^##^	−14.899	0.149	−35.328	5.531
High fat diet vs Regular chow^###^	4.747	0.642	−15.718	25.212
Fast vs Ab libitum^####^	11.747	0.254	−8.718	32.212
**AH**				
Female vs Male^#^	3.275	0.265	−2.572	9.122
GDX vs Sham^##^	1.641	0.574	−4.206	7.488
High fat diet vs Regular chow^###^	0.679	0.816	−5.179	6.536
Fast vs Ab libitum^####^	0.448	0.878	−5.409	6.305
**VMH**				
Female vs Male^#^	0.648	0.931	−14.245	15.540
GDX vs Sham^##^	8.102	0.279	−6.790	22.995
High fat diet vs Regular chow^###^	−7.679	0.305	−22.597	7.240
Fast vs Ab libitum^####^	−0.371	0.960	−15.290	14.548
**DMH**				
Female vs Male^#^	−3.817	0.308	−11.271	3.636
GDX vs Sham^##^	5.234	0.164	−2.220	12.688
High fat diet vs Regular chow^###^	6.381	0.092	−1.085	13.848
Fast vs Ab libitum^####^	2.766	0.459	−4.701	10.232

*^#^Here we use male mice as reference. ^##^Here we use sham mice as reference. ^###^Here we use regular chow as reference. ^####^Here we use fed mice as reference. B stands for regression coefficients. The p value is the smallest level α0 such that we would reject the null-hypothesis at level α0 with the observed data. The confidence interval (CI) is a range of values that’s likely to include a population value with a certain degree of confidence.*

## Discussion

One interesting observation of our study is that endogenous gonadal hormones are required to maintain normal PNNs in the ARH in both male and female mice. The ARH in the hypothalamus has been long believed to contain the first order neurons that respond to the peripheral signals, including leptin (Balthasar et al., 2004; Caron et al., 2018; Xu et al., 2018), insulin (Konner et al., 2007; Hill et al., 2010; Qiu et al., 2014), ghrelin (Wang et al., 2014), asprosin (Duerrschmid et al., 2017) and estrogen (Roepke et al., 2007; Xu et al., 2011; Herber et al., 2019). The ARH neurons include those expressing pro-opiomelanocortin (POMC) and those co-releasing agouti-related peptide (AgRP), neuropeptide Y (NPY), and GABA (referred to as AgRP neurons). POMC neurons synthesize and secret an anorexigenic peptide, α-melanocyte-stimulating hormone (α-MSH) which prevents overeating and body weight gain (Yaswen et al., 1999; Xu et al., 2005). On the other hand, AgRP neurons are orexigenic (Wu et al., 2009), and activation of AgRP neurons promotes eating even when mice are satiated (Aponte et al., 2011; Krashes et al., 2011). Recent evidence also indicates that non-AgRP GABAergic neurons in the ARH provide a redundant orexigenic mechanism to drive feeding and body weight gain (Zhu et al., 2020). Notably, the majority of PNNs in the ARH enmeshes AgRP neurons and GABAergic neurons, with only a small portion enmeshing POMC neurons (Mirzadeh et al., 2019). Interestingly, reduced PNNs in the ARH are found in ob/ob mice deficient in the leptin gene and in ZDF rats with a mutated leptin receptor gene (Mirzadeh et al., 2019; Alonge et al., 2020), highlighting an important role of leptin signaling in maintaining the normal PNNs in the ARH. Further, the central administration of FGF1 in ZDF rats can enhance PNNs in the ARH, associated with a prolonged glucose-lowering benefit (Alonge et al., 2020). Importantly, chemical disruption of PNNs significantly shortens this glucose-lowering effect of FGF1 (Alonge et al., 2020). Thus, PNNs in the ARH play an essential role in regulating glucose balance.

Here, we demonstrated that PNNs in the ARH are significantly reduced by removal of the ovaries in female mice, suggesting that PNNs are also regulated by ovarian hormones. Supporting this notion, co-administration of both ovarian hormones (estrogen and progesterone), which simulates pregnancy, can trigger PNN formation in the medial preoptic area, a hypothalamic region normally devoid of PNNs in non-pregnant females (Uriarte et al., 2020). While the role of progesterone in energy and glucose homeostasis is not clear (Wang and Xu, 2019), estrogen is well established to provide many metabolic benefits, including suppressing food intake, increasing energy expenditure, reducing body weight gain, enhancing insulin sensitivity, and lowering blood glucose (Drewett, 1973; Blaustein and Wade, 1976; Geary et al., 2001; Wallen et al., 2001; Riant et al., 2009; Rogers et al., 2009; Paladini and Roeper, 2014). Both ERα and estrogen receptor β (ERβ) are implicated in mediating these metabolic effects (Heine et al., 2000; Okura et al., 2003; Foryst-Ludwig et al., 2008). Notably, abundant ERα is expressed by POMC neurons in the ARH (Miller et al., 1995; Attia, 2010; de Souza et al., 2011), to a less extent by AgRP neurons (Olofsson et al., 2009; Roepke et al., 2011), while ERβ expression in the ARH is minimal (Merchenthaler et al., 2004). We recently showed that intact ERα signals are required for the glucose-sensing functions of ARH neurons (Yu et al., 2020). Further, estrogen can enhance glutamatergic synapses onto POMC neurons (Gao et al., 2007), and increase their excitability (Malyala et al., 2008; Saito et al., 2015). Importantly, we previously showed that selective deletion of ERα from POMC-lineage neurons in female mice results in hyperphagia and obesity (Xu et al., 2011), and attenuates estrogen-induced anorexia (Zhu et al., 2015). Here we observed that a portion of PNN-enmeshed ARH neurons co-express ERα and that PNNs in the ARH are reduced by ovariectomy. Collectively, these findings suggest that PNNs in the ARH may mediate, at least a portion of, estrogenic actions on energy and glucose homeostasis, and future investigations are warranted to test this possibility. Interestingly, the majority of PNN-enmeshed ARH neurons co-express androgen receptor, and we also observed similar reductions of PNNs in the ARH of male mice depleted with endogenous testosterone. Consistently, it has been reported that testosterone administration can stimulate PNN formation in the forebrain of song birds (Cornez et al., 2020). However, it is worth noting that testosterone can be converted into estrogen by aromatase (Henderson and Swanston, 1978). Thus, it remains to be tested whether the testosterone-induced PNN expression is mediated by testosterone *per se* or by converted estrogen.

Another hypothalamic region where PNNs are regulated is the TE, where female mice showed higher PNNs than males. Importantly, this sex difference was only observed in gonad-intact mice, but was blunted in mice with gonads surgically removed. These results suggest that PNNs in the TE may be also regulated by gonadal hormones. Supporting this possibility, estrogen has been shown to increase oxytocin binding in the TE of rats (Kremarik et al., 1995), indicating potential actions of estrogen in this region. It has to be pointed out that, unlike other hypothalamic regions (e.g., the ARH, VMH, etc.), the TE has received very little attention with no more than 5 publications regarding this nucleus available in the PubMed. Early neuroanatomic studies reported that cholecystokinin neurons and CGRP neurons are located in the TE (Lantos et al., 1995, 1996). Consistent with those studies, we found that a portion of PNN-enmeshed TE neurons co-express CGRP. In addition, the TE harbors nerve fibers that are immunoreactive for NPY, α-MSH, β-endorphin, corticortropin-releasing hormone, galanin, and substance *P* (Lantos et al., 1995, 1996), suggesting that the TE neurons receive a wide range of innervations and inputs from other neural populations. Interestingly, we found that in female mice only, PNNs in the TE were upregulated by chronic HFD feeding, while such effect was not observed after overnight fasting. It is worth noting that among many hypothalamic regions expressing PNNs, the TE is the only region where PNNs can be regulated by dietary interventions. Considering that the TE receives inputs from NPY and α-MSH, both of which are implicated in the regulation of feeding and body weight, we suggest that TE neurons and the PNNs enmeshing these neurons may play an under-appreciated role in the context of energy homeostasis. This possibility warrants future investigations.

In summary, here we systemically assessed PNNs in multiple hypothalamic regions and explored potential regulation by sex, gonadal hormones, dietary interventions, and their interactions. We acknowledge that the limitation of the current study is its descriptive nature. However, we want to point out that our results filled a gap of knowledge in the relevant field and provided a framework to develop new hypotheses and design future studies to determine the physiological functions of hypothalamic PNNs and to explore the regulatory mechanisms. For example, the strong regulation by gonadal hormones of PNNs in the ARH suggests that gonadal hormones may act through PNNs in the ARH to regulate energy and/or glucose homeostasis. Further, given that PNNs in the ARH are required to mediates metabolic benefits of FGF1 (Alonge et al., 2020), the efficacy of FGF1 may need to be examined in animals depleted of gonadal hormones, which would provide important pre-clinical data for potential applications of FGF1 or its analogs in aged men with testosterone deficiency or in post-menopausal women. In addition, the dietary regulation of PNNs in the TE suggests that this understudied hypothalamic nucleus may play a role in the regulation of feeding and body weight balance. Importantly, since this dietary regulation only existed in female mice but not in male mice, future studies need to include at least female animals, if not both. Only including male animals (a common practice in many laboratories) might miss potential discoveries about PNNs in the TE or TE neurons themselves.

## Data Availability Statement

The raw data supporting the conclusions of this article will be made available by the authors, without undue reservation.

## Ethics Statement

The animal study was reviewed and approved by the Baylor College of Medicine Institutional Animal Care and Use Committee.

## Author Contributions

NZ was involved in experimental design and most of procedures, data acquisition and analyses, and writing the manuscript. ZY assisted in data analysis. HaL, MY, YH, HeL, CL, LT, LW, NY, JH, NS, YY, and CW assisted in surgical procedures and production of study mice. L-LC and TZ were involved in the study design and manuscript preparation. YX was the guarantor of this work and, as such, had full access to all the data in the study and took responsibility for the integrity of the data and the accuracy of the data analysis. All authors contributed to the article and approved the submitted version.

## Conflict of Interest

The authors declare that the research was conducted in the absence of any commercial or financial relationships that could be construed as a potential conflict of interest.

## Publisher’s Note

All claims expressed in this article are solely those of the authors and do not necessarily represent those of their affiliated organizations, or those of the publisher, the editors and the reviewers. Any product that may be evaluated in this article, or claim that may be made by its manufacturer, is not guaranteed or endorsed by the publisher.

## Footnotes

1
www.empowerstats.com

2
http://www.R-project.org
