# Supplementary material for: Hypothalamic Perineuronal Nets Are Regulated by Sex and Dietary Interventions
Source: Front Physiol. 2021 Jul 28;12:714104. doi: 10.3389/fphys.2021.714104 (PMC8355523; doi:10.3389/fphys.2021.714104)
Supplement: Supplementary file 1 [file Data_Sheet_1.docx]

**Supplemental Table 1. Estrous stages of female sham mice at the time of perfusion.**

| Group | | Estrous Stage |
| --- | --- | --- |
| Female Sham | Chow fasting group | D to P |
|  |  | E to M |
|  |  | P |
|  | Chow ad libitum group | E |
|  |  | P |
|  |  | E to M |
|  | High fat diet fasting group | E |
|  |  | D |
|  |  | P |
|  | High fat diet ad libitum group | E |
|  |  | M |
|  |  | E |

**Supplemental Table 2. Effects of estrous stage on PNN fluorescence intensity in different hypothalamic areas.**

| Estrous stage | Proestrus | Estrus | Estrus to Metestrus | p* |
| --- | --- | --- | --- | --- |
| N | 3 | 4 | 2 |  |
| ARH (A.U.) | 102626.848 (73930.525-106510.770) | 65264.433 (51506.379-84484.494) | 114448.688 (108761.533-120135.842) | 0.194 |
| TE (A.U.) | 186013.837 (149454.355-190322.442) | 136123.755 (116011.756-154082.642) | 134106.806 (105143.453-163070.160) | 0.583 |
| PVH (A.U.) | 13223.234 (9018.510-15413.970) | 14414.107 (9523.421-18445.914) | 8063.304 (5810.137-10316.472) | 0.667 |
| PeFAH (A.U.) | 75262.621 (61808.037-77290.244) | 45206.772 (40013.605-66444.213) | 76742.507 (76421.758-77063.256) | 0.555 |
| LH (A.U.) | 287803.633 (264464.841-305340.897) | 291506.687 (287697.898-311386.380) | 371161.400 (356996.701-385326.099) | 0.170 |
| AH (A.U.) | 42935.262 (33908.943-44698.765) | 41568.843 (25421.749-56773.164) | 68308.849 (64502.329-72115.368) | 0.197 |
| VMH (A.U.) | 66497.795 (52472.350-114074.718) | 41263.094 (25864.638-59616.544) | 84368.413 (70969.549-97767.277) | 0.333 |
| DMH (A.U.) | 2628.126 (2566.827-4094.946) | 4022.097 (2169.154-6043.087) | 4414.175 (3199.731-5628.620) | 1.000 |

* The p value is calculated by Kruskal-Wallis one-way ANOVA analysis.

AH, anterior hypothalamus; ARH, arcuate hypothalamic nucleus; DMH, dorsomedial hypothalamic nucleus; LH, lateral hypothalamus; PeFAH, perifornical area of the anterior hypothalamus; PVH, paraventricular hypothalamic nucleus; TE, terete hypothalamic nucleus; VMH, ventromedial hypothalamic nucleus.

**Supplemental Table 3. Effects of estrous stage on the number of PNN-enmeshed cells in different hypothalamic areas.**

| Estrous stage | Proestrus | Estrus | Estrus to Metestrus | p* |
| --- | --- | --- | --- | --- |
| N | 3 | 4 | 2 |  |
| ARH | 106 (98-122) | 77 (65-98) | 105 (101-108) | 0.337 |
| TE | 63(62-66) | 67 (56-75) | 65 (58-71) | 0.962 |
| PVH | 28 (26-35) | 22 (18-23) | 33 (33-34) | 0.079 |
| PeFAH | 36 (34-43) | 32 (20-43) | 37 (31-43) | 0.607 |
| LH | 211 (210-219) | 216 (173-258) | 246 (241-250) | 0.607 |
| AH | 37 (35-40) | 43 (36-48) | 39 (37-41) | 0.741 |
| VMH | 102 (97-118) | 79 (65-95) | 93 (87-100) | 0.447 |
| DMH | 30 (26-34) | 33 (17-47) | 30 (21-40) | 1.000 |

* The p value is calculated by Kruskal-Wallis one-way ANOVA analysis.

AH, anterior hypothalamus; ARH, arcuate hypothalamic nucleus; DMH, dorsomedial hypothalamic nucleus; LH, lateral hypothalamus; PeFAH, perifornical area of the anterior hypothalamus; PVH, paraventricular hypothalamic nucleus; TE, terete hypothalamic nucleus; VMH, ventromedial hypothalamic nucleus.

**Supplemental Table 4. Metabolic parameters and fluorescence intensity of PNN in different hypothalamic areas in male and female mice.**

|  | Male | Female |
| --- | --- | --- |
| N | 25 | 24 |
| Cumulative intake (calorie) | 349.2 ± 46.1 | 332.1 ± 55.4 |
| Body weight change (g) | 2.8 (0.1-6.2) | 1.3 (-0.0-4.9) |
| Blood glucose change (mg/dL) | -8.5 (-16.5-14.5) | 22.0 (5.6-34.1) |
| Fat change (g) | 3.6 (1.4-7.4) | 1.0 (-0.7-4.2) |
| Lean change (g) | -0.8 (-1.8-0.0) | 0.6 (0.1-0.9) |
| FI of ARH (A.U.) | 58198.176 (40389.300-100766.152) | 81104.562 (40665.291-96911.804) |
| FI of TE (A.U.) | 121564.221 (73431.971-138938.932) | 145087.004 (122124.904-178074.892) |
| FI of PVH (A.U.) | 7872.184 (3982.502-10975.445) | 9209.487 (3456.496-12733.038) |
| FI of PeFAH (A.U.) | 53433.032 (30282.100-79810.191) | 51174.575 (38615.494-77867.471) |
| FI of LH (A.U.) | 243897.334 (217931.833-291721.598) | 283058.385 (220941.822-297422.600) |
| FI of AH (A.U.) | 39191.679 (30735.413-57673.875) | 44698.765 (29047.286-68913.723) |
| FI of VMH (A.U.) | 52459.061 (43700.578-63763.951) | 50772.674 (33587.993-67853.988) |
| FI of DMH (A.U.) | 6326.443 (4243.890-7637.352) | 3748.988 (2479.224-6747.313) |
| Number in ARH | 91 (75-112) | 96 (80-109) |
| Number in TE | 59 (47-70) | 63 (56-71) |
| Number in PVH | 16 (12-24) | 19 (12-25) |
| Number in PeFAH | 39 (26-45) | 36 (27-45) |
| Number in LH | 207 (179-219) | 210 (190-250) |
| Number in AH | 38 (33-41) | 43 (33-46) |
| Number in VMH | 94 (73-105) | 90 (71-103) |
| Number in DMH | 34 (24-43) | 29 (20-37) |

* Data are shown as mean ± SD if normally distributed and median (IQR) if not normally distributed. AH, anterior hypothalamus; ARH, arcuate hypothalamic nucleus; DMH, dorsomedial hypothalamic nucleus; FI, fluorescence intensity; LH, lateral hypothalamus; PeFAH, the perifornical area of the anterior hypothalamus; PNN, perineuronal nets; PVH, the paraventricular hypothalamic nucleus; TE, terete hypothalamic nucleus; VMH, ventromedial hypothalamic nucleus.

**Supplemental Table 5. Metabolic parameters and fluorescence intensity of PNN in different hypothalamic areas in sham and OVX female mice.**

|  | Sham | OVX |
| --- | --- | --- |
| N | 12 | 12 |
| Cumulative intake (calorie) | 321.2 ± 32.2 | 342.9 ± 71.6 |
| Body weight change (g) | 1.0 (-0.0-2.2) | 3.5 (-0.1-7.2) |
| Blood glucose change (mg/dL) | 13.2 (-12.8-22.0) | 30.5 (23.8-43.3) |
| Fat change (g) | 0.7 (-0.4-1.9) | 2.8 (-1.1-7.5) |
| Lean change (g) | 0.4 (-0.1-0.6) | 0.8 (0.5-1.0) |
| FI of ARH (A.U.) | 93632.1 (67232.9-103039.7) | 40295.5 (35500.4-87809.5) |
| FI of TE (A.U.) | 154706.6 (123358.3-181598.3) | 135691.9 (119879.6-162606.8) |
| FI of PVH (A.U.) | 10138.2 (4499.6-14023.7) | 7368.2 (3357.2-11712.7) |
| FI of PeFAH (A.U.) | 50633.4 (45165.4-76421.8) | 53051.1 (29060.7-81345.4) |
| FI of LH (A.U.) | 288510.7 (276840.0-327866.6) | 243013.3 (200801.4-284248.1) |
| FI of AH (A.U.) | 49582.2 (29047.3-70720.1) | 35424.3 (27489.9-63101.3) |
| FI of VMH (A.U.) | 51722.1 (35588.1-67854.0) | 50772.7 (33588.0-85427.2) |
| FI of DMH (A.U.) | 2786.5 (2375.5-5850.2) | 4048.9 (2943.3-8642.9) |
| Number in ARH | 106 (89-116) | 89 (72-106) |
| Number in TE | 63 (56-71) | 63 (58-72) |
| Number in PVH | 23 (13-29) | 17 (11-20) |
| Number in PeFAH | 30 (24-43) | 39 (35-57) |
| Number in LH | 232 (201-254) | 196 (190-219) |
| Number in AH | 42 (34-45) | 44 (32-47) |
| Number in VMH | 91 (77-103) | 87 (70-101) |
| Number in DMH | 27 (19-39) | 33 (23-37) |

* Data are shown as mean ± SD if normally distributed and median (IQR) if not normally distributed. AH, anterior hypothalamus; ARH, arcuate hypothalamic nucleus; DMH, dorsomedial hypothalamic nucleus; FI, fluorescence intensity; LH, lateral hypothalamus; OVX, ovariectomy; PeFAH, the perifornical area of the anterior hypothalamus; PNN, perineuronal nets; PVH, the paraventricular hypothalamic nucleus; TE, terete hypothalamic nucleus; VMH, ventromedial hypothalamic nucleus.

**Supplemental Table 6. Metabolic parameters and fluorescence intensity of PNN in different hypothalamic areas in sham and CAST male mice.**

|  | Sham | CAST |
| --- | --- | --- |
| N | 12 | 13 |
| Cumulative intake (calorie) | 351.9 ± 50.9 | 346.7 ± 43.5 |
| Body weight change (g) | 3.6 (1.0-6.6) | 2.6 (-0.4-5.5) |
| Blood glucose change (mg/dL) | -2.0 (-11.6-25.8) | -10.0 (-19.5-10.5) |
| Fat change (g) | 3.0 (1.6-6.6) | 4.8 (1.4-7.4) |
| Lean change (g) | 0.1 (-0.2-0.6) | -1.8 (-2.3--1.6) |
| FI of ARH (A.U.) | 93706.431 (51784.476-114942.509) | 50834.519 (38340.837-59915.213) |
| FI of TE (A.U.) | 105939.752 (61187.759-126083.000) | 131654.756 (89375.032-171116.398) |
| FI of PVH (A.U.) | 8512.097 (3017.583-12265.834) | 7556.147 (4952.196-9919.709) |
| FI of PeFAH (A.U.) | 43720.435 (27518.897-61917.945) | 66602.487 (38034.960-81870.506) |
| FI of LH (A.U.) | 254495.172 (192706.564-284616.370) | 234129.533 (227371.722-296580.458) |
| FI of AH (A.U.) | 32964.778 (28475.172-56548.442) | 41106.212 (34233.848-57673.875) |
| FI of VMH (A.U.) | 51796.785 (39781.536-61686.102) | 53668.756 (43856.835-63763.951) |
| FI of DMH (A.U.) | 5244.956 (2307.902-7866.622) | 6606.931 (4389.093-7366.663) |
| Number in ARH | 102 (76-122) | 87 (69-96) |
| Number in TE | 49 (47-65) | 66 (52-72) |
| Number in PVH | 16 (10-27) | 17 (14-21) |
| Number in PeFAH | 33 (25-44) | 39 (28-61) |
| Number in LH | 211 (187-219) | 190 (179-219) |
| Number in AH | 39 (35-41) | 38 (33-41) |
| Number in VMH | 83 (63-98) | 104 (82-106) |
| Number in DMH | 27 (17-35) | 36 (31-47) |

* Data are shown as mean ± SD if normally distributed and median (IQR) if not normally distributed. AH, anterior hypothalamus; ARH, arcuate hypothalamic nucleus; CAST, castration; DMH, dorsomedial hypothalamic nucleus; FI, fluorescence intensity; LH, lateral hypothalamus; PeFAH, the perifornical area of the anterior hypothalamus; PNN, perineuronal nets; PVH, the paraventricular hypothalamic nucleus; TE, terete hypothalamic nucleus; VMH, ventromedial hypothalamic nucleus.

**Supplemental Table 7. Metabolic parameters and fluorescence intensity of PNN in different hypothalamic areas in regular chow and high fat diet mice.**

|  | Regular chow | High fat diet |
| --- | --- | --- |
| N | 24 | 25 |
| Cumulative intake (calorie) | 309.8 ± 33.5 | 367.5 ± 49.3 |
| Body weight change (g) | 0.1 (-4.4-4.4) | 5.5 (-1.8-14.2) |
| Blood glucose change (mg/dL) | -11.4 (-50.0-47.3) | 27.5 (-19.5-124.0) |
| Fat change (g) | 0.0 (-1.9-4.0) | 6.4 (-3.2-12.4) |
| Lean change (g) | 0.3 (-3.2-1.7) | -0.0 (-3.0-1.6) |
| FI of ARH (A.U.) | 54427.784 (25514.979-125822.996) | 82302.765 (23893.590-119390.936) |
| FI of TE (A.U.) | 125059.958 (22150.380-194631.047) | 138208.327 (38654.342-212173.951) |
| FI of PVH (A.U.) | 6298.497 (884.846-24664.571) | 9152.010 (1322.480-57856.123) |
| FI of PeFAH (A.U.) | 51811.998 (12137.569-105662.680) | 52158.185 (12581.485-154202.569) |
| FI of LH (A.U.) | 267346.902 (127124.245-506953.399) | 275355.694 (126284.167-364158.542) |
| FI of AH (A.U.) | 37674.751 (6193.874-84055.297) | 47375.157 (5043.752-100817.849) |
| FI of VMH (A.U.) | 50152.773 (17798.986-270366.554) | 53614.133 (21081.542-159273.828) |
| FI of DMH (A.U.) | 3748.988 (457.674-17599.091) | 5586.737 (2029.354-10260.151) |
| Number in ARH | 102 (38-138) | 91 (58-146) |
| Number in TE | 62 (18-80) | 62 (27-102) |
| Number in PVH | 17 (3-38) | 19 (6-47) |
| Number in PeFAH | 36 (17-83) | 39 (15-123) |
| Number in LH | 209 (144-276) | 209 (149-289) |
| Number in AH | 37 (16-57) | 41 (13-61) |
| Number in VMH | 93 (46-158) | 87 (44-133) |
| Number in DMH | 27 (5-54) | 34 (8-59) |

* Data are shown as mean ± SD if normally distributed and median (IQR) if not normally distributed. AH, anterior hypothalamus; ARH, arcuate hypothalamic nucleus; DMH, dorsomedial hypothalamic nucleus; FI, fluorescence intensity; LH, lateral hypothalamus; PeFAH, the perifornical area of the anterior hypothalamus; PNN, perineuronal nets; PVH, the paraventricular hypothalamic nucleus; TE, terete hypothalamic nucleus; VMH, ventromedial hypothalamic nucleus.

**Supplemental Table 8. Metabolic parameters and fluorescence intensity of PNN in different hypothalamic areas in ad libitum and fast mice.**

| Nutrition status | Ad libitum | Fast |
| --- | --- | --- |
| N | 24 | 25 |
| Cumulative intake (calorie) | 350.2 ± 50.2 | 331.9 ± 51.6 |
| Body weight change (g) | 2.4 (0.1-5.6) | 2.4 (0.0-5.5) |
| Blood glucose change (mg/dL) | 16.5 (-14.2-37.9) | 2.0 (-11.5-27.5) |
| Fat change (g) | 3.0 (-0.1-6.0) | 1.9 (0.4-6.4) |
| Lean change (g) | 0.5 (-0.8-0.8) | -0.2 (-1.5-0.3) |
| FI of ARH (A.U.) | 69109.247 (39252.174-101231.326) | 72235.340 (41860.418-92257.357) |
| FI of TE (A.U.) | 138573.629 (81676.526-171262.479) | 124918.334 (103913.519-153739.198) |
| FI of PVH (A.U.) | 8512.097 (3954.150-12536.026) | 7873.411 (3478.612-11441.963) |
| FI of PeFAH (A.U.) | 56203.708 (34626.202-80158.666) | 50360.930 (34823.939-76725.930) |
| FI of LH (A.U.) | 282850.960 (192957.816-301467.407) | 257944.798 (227371.722-289217.715) |
| FI of AH (A.U.) | 43891.185 (28475.172-60814.826) | 38156.021 (31220.940-58725.193) |
| FI of VMH (A.U.) | 52918.208 (35893.170-61552.039) | 51134.509 (39336.531-66015.733) |
| FI of DMH (A.U.) | 6514.574 (2543.014-8461.656) | 4389.093 (2797.475-6606.931) |
| Number in ARH | 87 (66-111) | 94 (80-112) |
| Number in TE | 66 (50-72) | 60 (52-67) |
| Number in PVH | 17 (12-27) | 18 (14-24) |
| Number in PeFAH | 42 (25-59) | 35 (28-39) |
| Number in LH | 202 (176-223) | 211 (190-243) |
| Number in AH | 41 (33-44) | 40 (35-43) |
| Number in VMH | 88 (73-100) | 91 (72-106) |
| Number in DMH | 30 (20-39) | 32 (24-36) |

* Data are shown as mean ± SD if normally distributed and median (IQR) if not normally distributed. AH, anterior hypothalamus; ARH, arcuate hypothalamic nucleus; DMH, dorsomedial hypothalamic nucleus; FI, fluorescence intensity; LH, lateral hypothalamus; PeFAH, the perifornical area of the anterior hypothalamus; PNN, perineuronal nets; PVH, the paraventricular hypothalamic nucleus; TE, terete hypothalamic nucleus; VMH, ventromedial hypothalamic nucleus.

**Supplemental Figure 1**

**
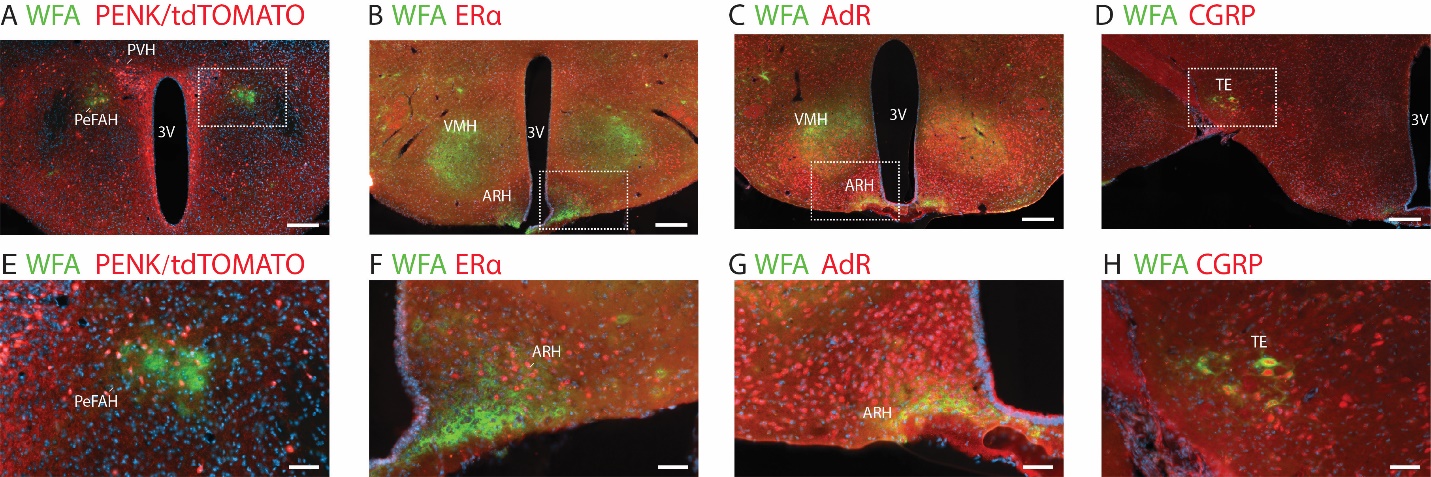
**

**Supplemental Figure 1.** Neurochemical identity of PNN-enmeshed neurons in the PeFAH, ARH and TE. (A and E) Representative microscopic images showing PENK-expressing neurons (red), WFA-labelled PNNs (green) and DAPI counterstaining (purple) in the PeFAH of a female mouse. (E) is higher magnification image of the PeFAH in (A). (B and F) Representative microscopic images showing ERα-expressing neurons (red), WFA-labelled PNNs (green) and DAPI counterstaining (purple) in the ARH of a female mouse. (F) is higher magnification image of the ARH in (B). (C and G) Representative microscopic images showing AdR-expressing neurons (red), WFA-labelled PNNs (green) and DAPI counterstaining (purple) in the ARH of a male mouse. (G) is higher magnification image of the ARH in (C). (D and H) Representative microscopic images showing CGRP-expressing neurons (red), WFA-labelled PNNs (green) and DAPI counterstaining (purple) in the TE of a female mouse. (H) is higher magnification image of the ARH in (D). ERα-positive neurons in the ARH surrounded by PNNs. Scar bar = 200 µm in (A-D). Scar bar = 50 µm in (E-H). 3V, 3^rd^ ventricle; AdR, androgen receptor; ARH, arcuate hypothalamic nucleus; CGRP, calcitonin gene-related peptide; ERα, estrogen receptor α; PeFAH, perifornical area of the anterior hypothalamus; PVH, paraventricular hypothalamic nucleus; TE, terete hypothalamic nucleus; VMH, ventromedial hypothalamic nucleus.
